# Investigation of *Cannabis sativa* Phytochemicals as Anti-Alzheimer’s Agents: An In Silico Study

**DOI:** 10.3390/plants12030510

**Published:** 2023-01-22

**Authors:** Nil Patil, Vaishnavi Chandel, Aarzu Rana, Mukul Jain, Prashant Kaushik

**Affiliations:** 1 Department of Life Sciences, Parul Institute of Applied Sciences, Parul University, Vadodara 391760, Gujarat, India; 2 Laboratory 209, Cell & Developmental Biology Laboratory, Centre of Research for Development, Parul University, Vadodara 391760, Gujarat, India; 3 Independent Researcher, 46022 Valencia, Spain

**Keywords:** Alzheimer’s disease, *Cannabis sativa*, AChE, BuChE, AutoDock, drug development

## Abstract

*Cannabis sativa* is a medicinal plant that has been known for years and is used as an Ayurvedic medicine. This plant has great potential in treating various types of brain diseases. Phytochemicals present in this plant act as antioxidants by maintaining synaptic plasticity and preventing neuronal loss. Cannabidiol (CBD) and Tetrahydrocannabinol (THC) are both beneficial in treating Alzheimer’s disease by increasing the solubility of Aβ42 amyloid and Tau aggregation. Apart from these therapeutic effects, there are certain unknown functions of these phytochemicals in Alzheimer’s disease that we want to elucidate through this study. In this research, our approach is to analyze the effect of phytochemicals in *Cannabis sativa* on multiple culprit enzymes in Alzheimer’s disease, such as AChE (Acetylcholinesterase), BChE (Butyrylcholinesterase), γ-secretase, and BACE-1. In this study, the compounds were selected by Lipinski’s rule, ADMET, and ProTox based on toxicity. Molecular docking between the selected compounds (THCV, Cannabinol C2, and Cannabidiorcol) and enzymes mentioned above was obtained by various software programs including AutoDock Vina 4.2, AutoDock, and iGEMDOCK. In comparison to Donepezil (BA = −8.4 kcal/mol, Ki = 1.46 mM), Rivastigmine (BA = −7.0 kcal/mol, Ki = 0.02 mM), and Galantamine (BA = −7.1, Ki = 2.1 mM), Cannabidiorcol (BA = −9.4 kcal/mol, Ki = 4.61 mM) shows significant inhibition of AChE. On the other hand, Cannabinol C2 (BA = −9.2 kcal/mol, Ki = 4.32 mM) significantly inhibits Butyrylcholinesterase (BuChE) in comparison to Memantine (BA = −6.8 kcal/mol, Ki = 0.54 mM). This study sheds new light and opens new avenues for elucidating the role of bioactive compounds present in *Cannabis sativa* in treating Alzheimer’s disease.

## 1. Introduction

*Cannabis *sativa L.** (*C. sativa*) is a dioecious, cross-pollinated, flowering plant that can grow to a height of between one and two meters. It is commonly known as hemp, cannabis, or marijuana [1]. *Cannabis sativa* has long been known to have both therapeutic and psychoactive properties. It is a multipurpose plant that is used for recreational, medicinal, and industrial purposes [2]. Cannabis is currently the focus of a significant amount of research due to its unique phytochemical constituents, i.e., secondary metabolites. The glandular trichomes in the bark, leaves, and especially the leaves of the female plant contain the most potent cannabinoid metabolites, which are known as phytocannabinoids [1,3]. Cannabis consists of a complex mixture of phytochemical compounds made up of flavonoids, terpenoids, cannabinoids [4], alkaloids, glycoproteins [5], and phytosteroids [6]. Over 565 cannabis constituents have been discovered in the cannabis plant [7], and over 150 compounds are considered phytocannabinoids [8,9,10]. The most investigated cannabinoids are Tetrahydrocannabinol (THC, which includes the two main components 8-THC and 9-THC), a major psychoactive component of cannabis, Cannabidiol (CBD), and cannabigerol (CBG), which are extracted from the resin formed by the female plants. [11,12].

Seven different classes are used to categorize the additional natural cannabinoids generated from *Cannabis sativa*, including Cannabinol (CBN), Cannabidiol (CBND), Cannabitriol (CBT), Cannabielsoin (CBE), Cannabichromene (CBC) Cannabicyclol (CBL), and other miscellaneous types [13]. To exert their many beneficial biological effects, Cannabinoids interact with a variety of receptors, including the cannabinoid receptors CB1 and CB2, as well as several additional non-cannabinoid receptors such as G-protein coupled receptors (GPR55, GPR3) and ion channels [14]. It has been suggested that one significant pathway of cannabis is used as an alternative treatment for many diseases, including chronic pain, nausea, multiple sclerosis, schizophrenia, glaucoma, sclerosis multiplex (SM), inflammatory bowel disorders (IBDs), nausea and vomiting, pain, appetite loss, epilepsy, [15] anxiety, Alzheimer’s disease, Parkinson’s disease, Huntington’s disease, and COVID-19. Phytocannabinoids, for example, can bind to these types of GPCRs, as shown in Table 1 [16,17].

Phytocannabinoids have gained significant attention due to their neuroactive, strong antioxidant, and anti-inflammatory properties. which may potentially treat different neurodegenerative diseases [38]. Cannabidiol (CBD) and 9-Tetrahydrocannabinol (THC) are two phytocannabinoids that associate with the endocannabinoid system (ECS), have antioxidant, anti-inflammatory, and neuroprotective characteristics, and can improve amyloid-β and NFT-related disorders as well as stimulate neurogenesis [39].

There is a need for a therapeutic drug that can fight against the culprit enzymes of Alzheimer’s disease (AD), which is pathologically characterized by the extracellular accumulation of aberrant amyloid-peptide peptides into plaques, the hyperphosphorylation of the microtubule-associated protein Tau resulting in the development of neurofibrillary tangles (NFTs) and neuroinflammation [40]. Preclinical research suggests that some cannabinoids, such as Tetrahydrocannabinol (THC) and Cannabidiol (CBD), may have pharmacological activities on the cholinergic system and amyloid-beta aggregation. These events all contribute to the irreversible and progressive neuronal dysfunction and cell death that cause cerebral atrophy [41]. Furthermore, researchers have been looking for new anti-AD drugs made from secretase enzyme inhibitors that specifically target secretases, such as BACE-1 and γ-secretase.

**Figure 1:**
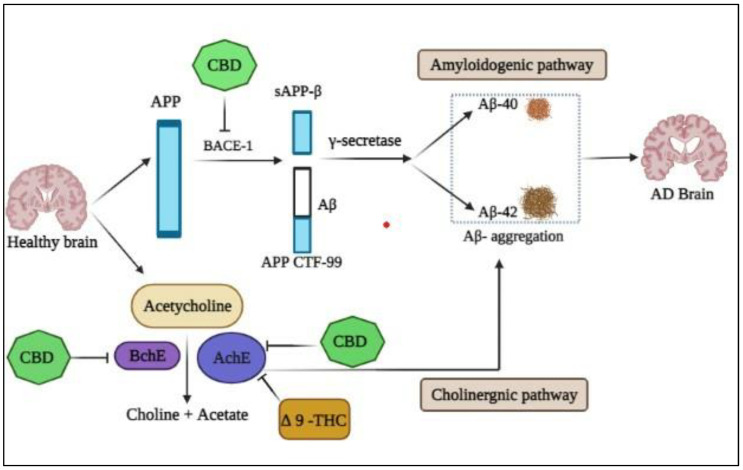
Inhibitory activity of Cannabis constituents on key pathways of Alzheimer’s disease. Δ-9-THC and CBD inhibits AChE and BChE. CBD also inhibits amyloid beta aggregate formation by inhibiting BACE-1.

These are the primary enzymes responsible for breaking down amyloid precursor protein (APP) into neurotoxic Aβ fragments (Aβ42) [42,43,44,45,46]. However, the medications employed in this treatment have limitations and adverse effects, such as nausea and vomiting, and have no impact on the processes that may contribute to AD, such as amyloid genesis, reactive oxygen species, and neuro-inflammation (Figure 1) [47,48,49]. Plant-based therapeutics for AD are the focus of more research because they have fewer side effects than synthetic medications. In this study, we used in silico methods to examine the effects of a panel of cannabinoids on AChE, BuChE, and BACE-1 activities, including CBD, THC, cannabigerol (CBG), cannabigerolic acid (CBGA), cannabibicitran (CBT), cannabidivarin (CBDV), cannabichromene (CBC), and Cannabinol (CBN).

The effectiveness of psychoactive cannabinoids in the treatment of psychiatric manifestations, particularly agitation and aggression from AD, cannot be conclusively determined from the clinical trials examined. Two factors have significantly hampered the achievement of conclusive results: (1) polypragmasia, which is characterized by the use of established or less established psychotropic drugs (other than cannabinoids) to reduce agitation and aggressive behavior in patients; and (2) a large number of concomitant symptoms, such as pain (which frequently causes anxiety and agitation) [50].

## 2. Results

### 2.1. Drug Likeliness Properties Analysis

Drug development is an expensive and drawn-out process. With the aid of computational methods, it is now easier to predict the elements that define a compound’s therapeutic potential. Drug-relevant parameters include CLogP, solubility, molecular weight (MW), topological molecular polar surface area (TPSA), etc. The OSIRIS Property Explorer was used to forecast these. OSIRIS accurately predicted the toxicity for 58.66% of compounds that were not harmful and 41.3% of compounds from 75 compounds that were potentially harmful. Lipinski’s rule of five is often employed by pharmaceutical chemists in drug design and development to determine the oral bioavailability of possible lead or therapeutic compounds (Supplementary Table S1). The Lipinski rule, Veber rule, Ghose filter, lead likeness, and PAINS (Pan Assay Interference Compounds) were followed by 58.6% of the compounds (44 compounds). Those 44 compounds were further analyzed for pharmacokinetic properties through the SWISS-ADME server for consideration as a drug.

### 2.2. ADMET Analysis

**Figure 2:**
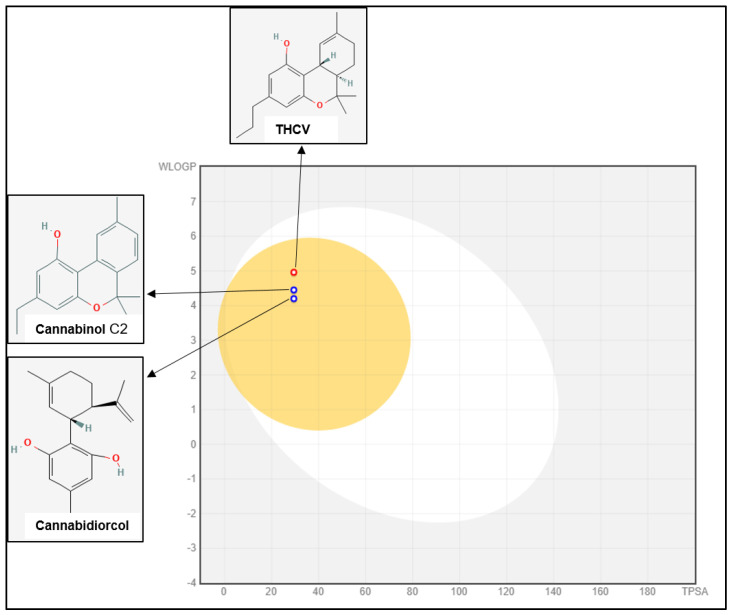
Predicted boiled-egg plot (Swiss ADMET) of THCV, Cannabinol C2 and Cannabidiorcol. The white region (HIA) predicts the physicochemical space of molecules that are able to be absorbed by the gastrointestinal system, while the yellow region (BBB) predicts the physicochemical space of molecules that are able to penetrate the brain.

SWISS-ADME is an online server that lets users create their own ligands or drug molecules and incorporate SMILES data from PubChem. It includes properties such as lipophilicity, water solubility, Log S, and medicinal chemistry. Using five distinct Lipinski, Ghose, Veber, Egan, and Muegge criteria, the SWISS-ADME section provides the physicochemical characteristics of potential oral medication candidates (Table 2). The in silico ADMET prediction of selected compounds such as eucalyptol and Cannabinol C2 shows 93.96 and 96.505 absorption with BBB permeability (0.592), respectively, despite the fact that they were not expected to act on p-glycoprotein (Figure 2). Another essential factor in distribution is distribution volume, which describes how medications are distributed in different tissues in vivo. Two of the most crucial enzyme systems in the liver for drug metabolism are the cytochrome P450s (CYP2D6 and CYP3A4). The findings show that none of the substances tested will be broken down by the cytochrome P450 enzymes. In terms of toxicity, CBD derivatives might not have an AMES or hepatotoxicity profile and might not block the hERG channel.

### 2.3. Toxicity Analysis

The projected acute oral toxicity (LD50) of the substances ranged from the least lethal Cannabinol C2 (1310 mg/kg) to THCV (482 mg/kg). The compounds had LD50 values in class IV, indicating that they were not toxic if swallowed. These selected compounds were further compared with the already available AChE and BuChE inhibiting drugs, and of all three analogs, the Cannabinol-C2 ligand could be used in drug development as it possesses an LD50 value that is even lower, thereby making it an efficient candidate.

### 2.4. Molecular Docking

**Figure 3:**
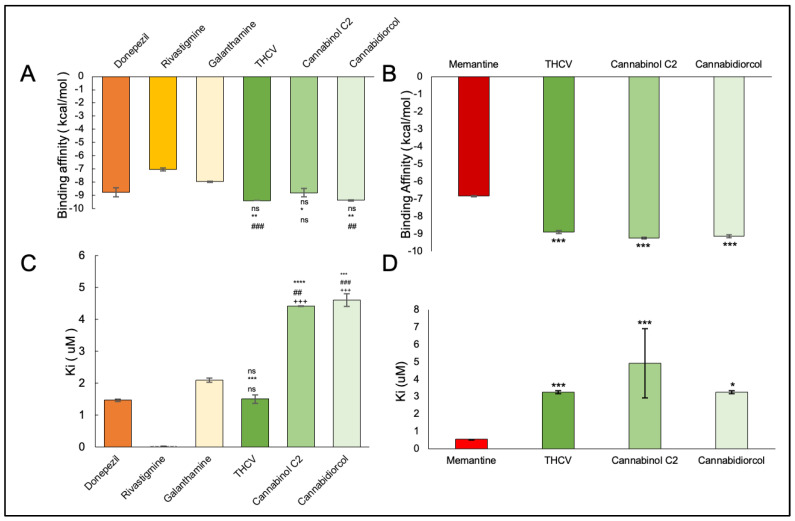
Graphical representation showing comparison of binding affinity and inhibition constant among (**A**,**C**) AChE inhibitors (Donepezil, Rivastigimine, Galantamine) and THCV, Cannabinol C2 and Cannabidiorcol (**B**,**D**) Butyrylcholinesterase inhibitors (Memantine) and THCV, Cannabinol C2 and Cannabidiorcol (* *p* < 0.1, ** *p* < 0.01, *** *p* < 0.001) and values are mean ± standard deviation where *p* * (Donepezil), *p* # (Rivastigmine), *p* + (Galantamine) and *p* * (Memantine).

**Figure 4:**
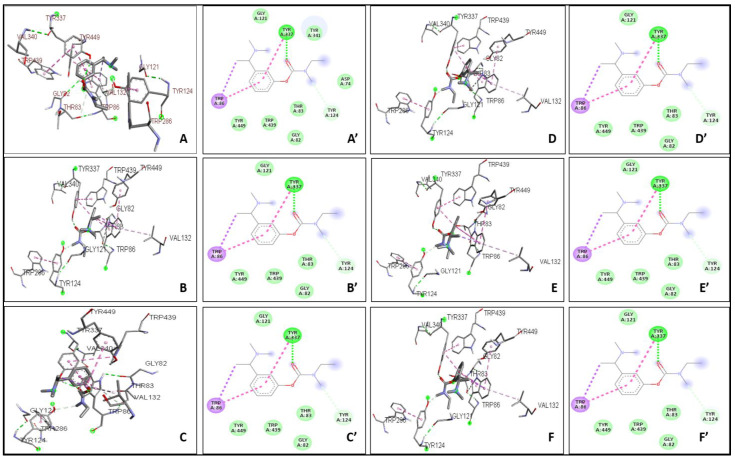
Interaction of Acetylcholinesterase with drugs (**A**–**C’**) and compounds (**D**–**F’**) (**A**) 3D interaction of Galantamine. (**A’**) 2D interaction of Galantamine. (**B**) 3D interaction of Donepezil. (**B’**) 2D interaction of Donepezil. (**C**) 3D interaction with Rivastigmine. (**C’**) 2D interaction with Rivastigmine. (**D**) 3D interaction with THCV. (**D’**) 2D interaction with THCV. (**E**) 3D interaction with Cannabinol C2. (**E’**) 2D interaction with Cannabinol C2. (**F**) 3D interaction with Cannabidiorcol. (**F’**) 2D interaction with Cannabidiorcol.

**Figure 5:**
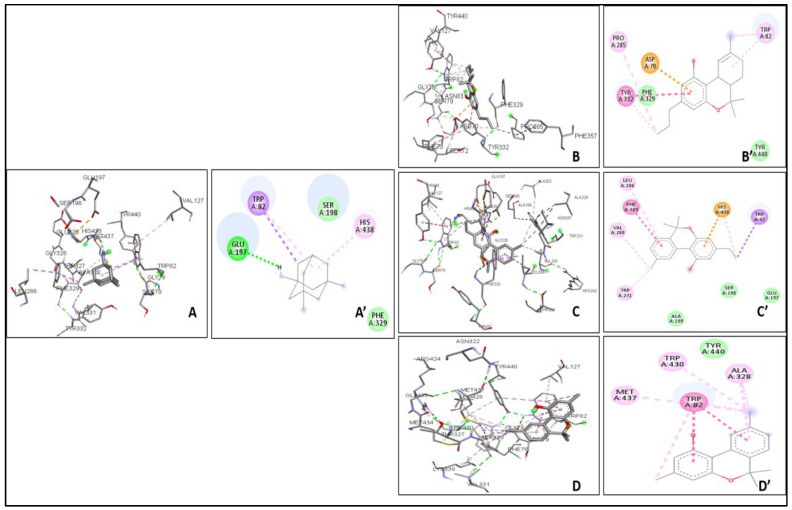
Interaction of Butyrylcholinesterase with drugs (**A**,**A’**) and compounds (**B–D’**) (**A**) 3D interaction with Memantine. (**A’**) 2D interaction with Memantine. (**B**) 3D interaction with THCV. (**B’**) 2D interaction with THCV. (**C**) 3D interaction with Cannabinol C2. (**C’**) 2D interaction with Cannabinol C2. (**D**) 3D interaction with Cannabidiorcol. (**D’**) 2D interaction with Cannabidiorcol.

The binding affinity between the ligand and the enzyme was identified by using AutoDock Vina 4.2, AutoDock, and iGEMDOCK. The Ki of the ligands were found with the help of AutoDock. The ΔG, Van der Waals, and hydrogen bond energies were calculated using iGEMDOCK. The ligands that were used are THCV, Cannabinol C2, and Cannabidiorcol. The binding interactions and Ki for ligands docked against AChE are summarized in Table 3. Galanthamine, Donepezil, and Rivastigmine all had binding affinities of −8.4, −7.0, and −7.1 kcal/mol, respectively. THCV and Cannabidiorcol had the highest binding affinity (−9.4 kcal/mol for both) compared to Cannabinol C2 (−9.3 kcal/mol). Donepezil, Rivastigmine, and Galantamine are the drugs taken against AChE. The binding affinity obtained for Memantine is −6.8 kcal/mol, which is taken against butyrylcholinesterase. Among all three ligands, the highest binding affinity obtained was for Cannabinol C2 and Cannabidiorcol (−9.2 kcal/mol), which is much higher than that of Memantine. The Ki obtained for Donepezil, Rivastigmine, and Galantamine were 1.46, 0.02, and 2.1, respectively. The Ki of standard drugs was less than that of the ligands taken, which signifies that the inhibitor has more potency against any enzyme or protein. The Ki of the ligands, i.e., THCV, Cannabinol C2, and Cannabidiorcol, were 1.50, 4.42, and 4.61, respectively. Memantine was the standard drug taken against Butyrylcholinesterase, which gave a Ki of 0.54, which is much less compared to the compounds taken i.e., THCV, Cannabinol C2 and Cannabidiorcol of Ki 3.25, 4.32, and 3.26, respectively (Figure 3). The interactive amino acids were visualized using the BIOVIA Discovery Studio. The 2D and 3D structure of the interactions is shown in Figure 4 and Figure 5. The interactive amino acids of THCV and Cannabidiorcol were similar, i.e., VAL340, TYR337, TRP439, TYR449, GLY82, THR83, TRP86, VAL132, GLY121, TYR124, and TRP286; whereas the drugs differed in their interactive amino acid residues in the AChE enzyme. For BuChE, the interactive amino acids for Memantine GLU197, TYR440, VAL127, GLY78, SER79, VAL331, TYR332, PHE329, THR327, ALA328, GLY326, LEU286, GLU125, SER198, HIS438, MET437. THCV contained amino acids that are TYR440, VAL127, TRP82, GLY78, ASN83, SER79, PHE329, PRO285, PHE357, TYR332, SER72, PHE73, ASP70; for Cannabinol C2 it was TYR440, VAL127, GLU197, ALA202, ALA229, ASN397, TRP231, ALA199, SER198, GLU325, HIS438, TRP82, GLY78, SER79, PHE329, TYR332, GLY326, LEU286, THR204, ARG242, VAL228 and for Cannabidiorcol GLY78, GLY435, ASN322, TYR440, VAL127, ARG424, MET437, LEU428, MET434, THR327, LYS339, VAL331, TRP430, ALA328, PHE76, TRP82. The target proteins (AChE and Butyrylcholinesterase) and compounds (THCV, Cannabinol C2, and Cannabidiorcol) were re-docked with iGEMDOCK (version 2.1). iGEMDOCK finds distinct bond energies that occur between proteins and chemicals, such as hydrogen bonds (H-Bond), Van Der Waals (VDW) interactions, and electrostatic interactions. The docking data showed that Cannabinol C2 had the lowest docking energy (−96.44 kcal/mol) in comparison to AChE, and the highest docking energy (−81.229 kcal/mol) in comparison to Butyrylcholinesterase. As shown in Table 4, Cannabinol C2 had the highest VDW (−81.229 kcal/mol) and the lowest VDW (−93.67 kcal/mol) for AChE and BuChE, respectively. The binding scores obtained using these tools might not be an exact representation of the true binding affinities, but they could be helpful in describing the relative affinities of two or more ligand molecules bound to the same or to different binding sites.

## 3. Discussion

Numerous health advantages of cannabinoids have been recognized, including neuroprotective properties against neurodegenerative disorders. Cannabinoids have been linked to a variety of helpful pharmacological benefits on the one hand, and toxic and unpleasant consequences on the other. According to a recent study, cannabinoid dosage and consumer age have a direct impact on health [51].On the contrary, growing scientific data suggests that CBDs and their changing tone might be viable therapeutic approaches for treating AD. Therefore, the study of AD related pathways and their respective enzyme regulation studies play a significant role. Cannabinoids have been shown to lower oxidative stress and excitotoxicity, as well as the production of amyloid plaques and neurofibrillary tangles [52]. CBD also plays a vital role in AD therapeutics in several manners, such as heat shock proteins and ubiquitin-conjugating enzymes, which are essential regulators of autophagy and are encoded by genes that CBD increases at the mRNA level [53]. The pharmacological effects of CBD on autophagy in AD were validated by a recent study that examined autophagic alterations brought on by prolonged CBD administration in 6-month-old APP/PS1 mice [54]. Additionally, CBD inhibits the hyper phosphorylation of glycogen synthase kinase 3 (GSK-3) brought on by Aβ and might be a novel treatment option for AD [55]. CBD does not only interact with cannabinoid receptors in the endo cannabinoid system. Instead, it is highly pleiotropic and acts on a variety of other receptors, including adenosine, glycine, non-endocannabinoid G protein-coupled, serotonin (5-HT), opioid, nicotinic acetylcholine, and transient potential Vanilloid receptor type 1 (TRPV1) receptors (i.e., enzymes and ion channels) [56]. CBD has several advantageous features that have been demonstrated in research due to its remarkable diversity of modes of action. These include qualities that are anti-oxidative, anti-inflammatory, analgesic, anticancer, anticonvulsant, anxiolytic, antidepressant, in addition to others [57].

The immunomodulatory impact of the CB2 receptor, which controls microglial activity, can reduce AD neuroinflammatory processes [58]. Another important effect is on acetylcholine availability and the inhibition of AChE-induced aggregation. BACE-1 and γ-secretase, which cleaves APP and forms amyloid beta oligomers that mediate cholinergic neurotransmission, is a major enzyme responsible for pathological alterations in AD; this dysfunction is directly related to neuroinflammation and cholinergic insufficiency in the CNS. Recent research indicates that *Cannabis sativa* secondary metabolites, CBD, activate PPARγ via the cell signaling Wnt/β-catenin pathway [59]. This activation reduces the neurotoxic nature of Aβ amyloids and oxidative stress in PC12 cells. CBD also increases cell viability and decreases ROS levels with the reduction in the peroxidation of lipids. There is also a decrease in Tau hyper phosphorylation and the inhibition of AChE. According to some in vitro studies, CBD increases the Aβ amyloid protein’s proteolytic pathway on SHY5YAPP+ cells (a neuroblastoma cell line) by inducing APP ubiquitination, which boosts cell viability [60]. Ethyl, which is a functional group in cannabinol C2 and is similar to Galantamine, can effectively enter the active pocket of AChE [61].Furthermore, THCV consist of Enol as a functional group that might be identified to break up amyloid plaques and enhance the rapid clearance of toxic aggregates in AD which is relatively similar to keto-enol pharmacophores [62].

In a recent study, THCV shows a significant effect at 50 uM and a decreased epileptiform burst speed in mice brain [63]. In M Sprague–Dawley rats/CB2 knockout mice, THCV also improved motor activities, reduced neuronal loss and reduced microglial activation [64]. Another analog, Cannabidiorcol, has shown promising results (LC50 = 0.348 ± 0.002 μg/mL), similar to vincristine sulfate in a cytotoxicity assay relating to cancer [65]. In this study, the interactive molecule of compounds (e.g., the Enol and keto groups) were showing significant binding with AChE and BuChE [66]. Our study demonstrates that the previous in vitro analyses were correlated with our in silico, approach and provided an additional step toward its validation as a putative inhibitor for AD.

## 4. Materials & Methods

The in silico analysis involved screening CBD and its 74 analogs for their binding and interaction with AChE and BuChE receptors. The ligands were visualized for their physiochemical properties, including pharmacokinetic properties and drug-likeness properties, as well as for their consideration as drugs for AChE and BuChE regulation. A detailed description of each part of the methodology is provided below:

### 4.1. Retrieval of the Ligand Molecule and Protein Structure for ADME Studies

#### 4.1.1. Protein Preparation

The crystal three-dimensional structure of the target receptors AChE (PDB ID: 4PQE), gamma secretase (PDB ID: 6IYC), BACE-1 (PDB ID: 1SZG), and BuChE (PDB ID: 4BDS) was retrieved from PDB https://www.rcsb.org/ (accessed on 11 November 2022) and used as a rigid receptor. Auto Dock MGLTools was used to prepare the receptor protein. The deposited protein data consisted of water molecules and NAG- and NAM-like molecules to stabilize the crystallographic structure of the proteins. The enzymes were prepared by removing the NAG, NAM, and water molecules from the sequence to be used for further study, assigning bond orders, adding hydrogen atoms, and distributing Kollmann’s charge equal to the whole receptor. The energy minimization of protein was done through the Chiron webserver https://dokhlab.med.psu.edu/chiron/login.php (accessed on 11 November 2022) [67].

#### 4.1.2. Ligand Preparation

The chemical structures of known inhibitors for AChE, γ secretase, BACE-1, and BuChE were retrieved from the Drug Bank database, and other compounds described in this study were mainly CBD and its 74 derivatives. Those analogs were drawn and retrieved from ChemDraw (https://chemdrawdirect.perkinelmer.cloud/js/sample/index.html (accessed on 13 November 2022) and PubChem https://puBuChEm.ncbi.nlm.nih.gov/ (accessed on 13 November 2022). After retrieval, the chemical compounds were energy minimized through Avogadro software 1.2.0 [68].

### 4.2. Drug Likeliness Properties

The drug-likeness, mutagenic, tumorigenic, reproductive, and irritating impacts of drug-toxicity risk factors were examined using the OSIRIS Data Warrior V5.2.1 program. It was used to study the toxicological characteristics of compounds that are possible orally active therapeutic candidates in clinical applications. An orally active compound should follow the Lipinski rule with zero violations. The “rule of five” model proposed by Lipinski suggested that for a compound to be consider orally bioactive, the violation of two or more of these conditions predicted a molecule to be a non-orally available drug [69].

### 4.3. ADME and Toxicity Test

#### 4.3.1. ADME Properties

ADME is required to assess the pharmacodynamics of a suggested chemical that might be employed as a medicine. The SWISS-ADME server (http://www.swissadme.ch/, accessed on 29 November 2022) was used to analyze the chosen ligands retrieved from PubChem and ChemDraw using canonical SMILES. As an input system, the structural data file and simplified molecular data input format was utilized to calculate the absorption, distribution, metabolism, excretion, and toxicity (ADMET) values. SWISS-ADME allows the user to study parameters such as lipophilicity, water solubility, Log S, drug likeness rules, and some medicinal chemistry. The observed values of those compounds are presented in Table S1. Through their pharmacokinetic properties, 44 compounds were shown to follow Lipinski’s rules [70]. Out of those 44 compounds, seven were able to cross the BBB (Blood-Brain Barrier) [71] and were further evaluated for toxicity determination. Out of those seven compounds, only three (THCV, Cannabinol-C2, and Cannabidiorcol) were able to cross the CNS and were studied further.

#### 4.3.2. Toxicity Prediction

Toxicology prediction helps predict the tolerability of a chemical before it is employed in a human or animal model. Computer-based methods are now available for obtaining a safety profile of the required substance to quantify toxicity. To analyze the hazardous effects of those three compounds, the Protox-II website https://tox-new.charite.de/protoxII/ (accessed on 16 November 2022) was utilized with LD50, Hepatoxicity, Cytotoxicity, and Immunotoxicity as toxicity parameters of a query chemical molecule that are predicted with this server [72].

### 4.4. Molecular Docking

The interaction between proteins (AChE and BuChE) and ligands (CBD and its 74 analogs) was determined by conducting molecular docking using AutoDock 4, AutoDock Vina 4.2, and iGEMDOCK in a triplicate manner. For the specific docking, the active site of AChE and BuChE was determined using CASTp 3.0 http://sts.bioe.uic.edu/castp/ (accessed on 14 November 2022). Kollmann’s charges, Gasteiger partial charges, and polar hydrogen atoms were added to the proteins. To achieve the best conformational docking results, the grid box was centered on the binding site of the ligand and the auto grid position was set at (x: −27.195, y: 21.246, z: −10.429) for AChE and at (x: 135.922, y: 140.041, z: 43.131) for BuChE. Gamma secretase and BACE-1 showed non-significant results with Hecogenin and Imatinib, respectively, so they were not used in further research (Supplemental Figure S3). The docking algorithm with 100 runs had the Lamarckian genetic algorithm (LGA) and the empirical free energy function as default parameters. For iGEMDOCK the population size was 200, there were 70 generations, and the number of solutions was two with the standard docking setting. After generating a set of poses, the best fit was selected, which represented the total binding energy in the form of hydrogen bonds (HB), van der Waals forces (VDW), and electrostatic interaction. BIOVIA Discovery Studio v. 21.1.0 was used to display the docked conformations and 3D target-ligand interactions, which assisted in analyzing and predicting the kind of amino acid involved and their interactions. Donepezil, Rivastigmine, and Galantamine, which had binding affinities of −8.77 kcal/mol, −7.0 kcal/mol, and −7.97 kcal/mol, respectively, were chosen as a reference to compare with analogues for the AChE receptor [73]. Memantine had a binding affinity of −6.83 kcal/mol and was chosen as a reference to compare with analogues for the BuChE receptor [74,75].

## 5. Conclusions

In comparison to known drugs, THCV, Cannabinol C2 and Cannabidiorcol dominated cannabinoids’ inhibitory activities on AChE and BuChE. Computational approaches suggest that THCV, Cannabinol C2, and Cannabidiorcol are more appropriate for the inhibition of the enzymes AChE and BuChE, which act as the culprits of Alzheimer’s disease. Cell and animal studies are needed to improve the efficacy of these cannabinoids and to learn more about the effecting pathways.

## Figures and Tables

**Table 1:** Major metabolites reported in extracts of *C. sativa* which affects the AD pathway and its medicinal characteristics.

Compound Class & Plant Tissue Type	Name	Effects on AD (In Vivo)	Effects on AD (In Vitro)	Precursor	Medicinal Characteristics [3,18]	References
Neutral cannabinoids (Trichomes, Female flowers, Roots/Apoplast (secretion pathway))	Cannabidiol (CBD)	Male Wistar rats utilise it as a Streptozotocin (STZ)- induced AD model, CBD enhances the brain glucose metabolism. Activation of the PPARγ via Wnt/β-catenin pathway	Pretreatment restores the synaptic transmission that was reduced by Aβ in a C57 mouse hippocampal slice.	CBGA	Anti–fungal and anti–bacterial against methicillin resistant strains, sedative and analgesic potential and anti–epileptic potential	[19,20,21,22,23,24,25]
	Tetrahydrocannabinol(THC)	THC reduces the Aβ burden in 5XFAD/APP mice	Compared to untreated controls, transgenic Tg4-42 mice expressing human A4-42 showed less neuronal death.	CBGA	Psychotropic & psychoactive properties	[20,26,27,28]
	Cannabichromene (CBC)	CBC (10–75 mgkg^−1^ i.p. per day) significantly decreased motor activity in a model of electroshock seizure during the first 10 min interval, but only the maximum dose was beneficial.	in vitro CBC improved the viability of neural stem cells	CBGA	Anti–inflammatory, sedative and analgesic potential	[29,30,31,32]
	Cannabidivarin (CBDV)	Inhibits oxytosis and prevents loss of energy in HT22 cells (50% inhibition at 1.1 μM and 90 nM, respectively), as well as reducing Aβ toxicity (50% inhibition at 100 nM) and trophic withdrawal (50% inhibition 350 nM);	Prevents oxytosis in Ht22 cells (mouse hippocampal cell) MC65 cells (human nerve cell line)	CBGVA	_	[30,33,34]
	Cannabicyclol (CBL)	_	_	CBC	_	[20,33]
	Cannabinol (CBN)	Inhibiting oxytosis and prevent loss of energy in HT22 cells (50% inhibition at 1.1 μM and 90 nM, respectively), as well as reducing Aβ toxicity (50% inhibition at 100 nM) and trophic withdrawal (50% inhibition 350 nM);	Along with its capacity to promote the breakdown and clearance of pre-formed A aggregates in MC65 cells at a concentration of 100 nM in HT-22 cells and cortical embryonic E18 neurons	THC	Mild psychoactive potential	[30,33]
	Cannabidiphorol (CBDP)	_	_	_	Antinociceptive,	[35]
	Tetrahydrocannabivarin (THCV)	_	_	CBGVA	Anti–dyskinesia in Parkinson’s disease	[35]
Flavonols (leaves, stems, seeds/lypophyl nature suggest cellular retention)	Cannabigerol (CBG)	Retains trophic factors present in cortical neurons of rat (effective concentration 50% = 1.5 μM) and inhibits the oxytosis in nerve cells (HT22) of mouse	Prevents oxytosis in Ht22 cells (mouse hippocampal cell MC65 cells (human nerve cell line)	OLA, GPP	Analgesic, Anti–inflammatory, Anti–Cancer, Psychotropic, Psychoactive	[30,34,36]
	Canniflavin A	_	Exhibits anti-inflammatory activity	Chrysoeriol	_	[32,36,37]

**Table 2:** Analysis of Pharmacokinetic properties and Protoxicity of three CBD analogues.

Compound	Mw	HA	HD	Absorption	Lipinski’s Rule Violation	Solubility	BBB Permeability	CNS Permeability	CYP2D6	LD50 (mg/kg)	Toxicity Class
Cannabidiorcol	254.3	2	1	92.851	0	−4.599	0.397	−1.368	NO	800	4
THCV	286.4	2	1	91.821	0	−4.403	0.336	−1.99	NO	482	4
Cannabinol C2	268.4	2	1	93.96	0	−4.834	0.5	−1.32	NO	1310	4

**Table 3:** Comparison of different docking parameters between three selected CBD analogues with AChE and BChE with reference drugs.

	AChE				BChE			
Compounds	B.A. (kcal/mol)	H bonds	Ki (μM)	Interactive Amino Acids	B.A. (kcal/mol)	H bonds	Ki (μM)	Interactive Amino Acids
THCV	−9.4	1	1.50	VAL340, TYR337, TRP439, TYR449, GLY82, THR83, TRP86, VAL132, GLY121, TYR124, TRP286	−8.9	0	3.25	TYR440, VAL127, TRP82, GLY78, ASN83, SER79, PHE329, PRO285, PHE357, TYR332, SER72, PHE73, ASP70
Cannabinol C2	−9.3	1	4.42	VAL340, TYR337, TRP439, TYR449, GLY82, THR83, TRP86, VAL132, GLY121, TYR124, TRP286	−9.2	3	4.32	TYR440, VAL127, GLU197, ALA202, ALA229, ASN397, TRP231, ALA199, SER198, GLU325, HIS438, TRP82, GLY78, SER79, PHE329, TYR332, GLY326, LEU286, THR204, ARG242, VAL228
Cannabidiorcol	−9.4	2	4.61	VAL340, TYR337, TRP439, TYR449, GLY82, THR83, TRP86, VAL132, GLY121, TRP286, TYR124	−9.2	1	3.26	GLY78, GLY435, ASN322, TYR440, VAL127, ARG424, MET437, LEU428, MET434, THR327, LYS339, VAL331, TRP430, ALA328, PHE76, TRP82
Memantine	-	-	-	-	−6.8	1	0.54	GLU197, TYR440, VAL127, TRP82, GLY78, SER79, VAL331, TYR332, PHE329, THR327, ALA328, GLY326, LEU286, GLU125, SER198, HIS438, MET437
Donepezil	−8.4	2	1.46	TYR337, TRP439, TYR449, VAL340, GLY82, THR83, GLY121, TRP86, VAL132, TRP286, TYR124	-	-	-	-
Rivastigmine	−7.0	2	0.02	TYR449, TRP439, TYR337, VAL240, GLY482, THR83, VAL132, TRP86, GLY121, TRP286, TYR124	-	-	-	-
Galantamine	−7.1	0	2.1	TYR337, TYR449, VAL340, TRP439, GLY82, THR83, TRP86, TYR124, GLY121, VAL132	-	-	-	-

**Table 4:** Docking energies of compounds with AChE and BChE receptors by iGEMDOCK.

Compounds	AChE			BChE		
	TE	VDW	HB	TE	VDW	HB
THCV	−91.33	−87.579	−4.5	−83.95	−78.95	−5
Cannabinol C2	−96.44	−93.67	5	−81.229	−81.229	0
Cannabidiorcol	−89.76	−86.579	−4.5	−78.94	−74.3765	−4.5

## Data Availability

Not applicable.

## Supplementary Materials

The following supporting information can be downloaded at: https://www.mdpi.com/article/10.3390/plants12030510/s1, Figure S1: 3D & 2D representation of BACE-1 & Gamma Secretase with inhibitors Hecogenin and Imatinib respectively; Table S1: All 75 compounds ADMET properties; Table S2: Compounds passing HHP and Lipinski’s rule; Table S3: Compounds passing BBB permeability.

## Abbreviations

**CBD**	Cannabidiol
**THC**	Tetrahydrocannabinol
**AChE**	Acetylcholinesterase
**BuChE**	Butryrylcholinesterase
**AD**	Alzheimer’s disease
**γ-** **secretase**	Gamma-secretase
**BACE-1**	Beta secretase-1
**ADMET**	Absorption, Distribution, Metabolism, Excretion and Toxicity
**THCV**	Tetrahydrocannabivarin
**iGEMDOCK**	A Graphical Environment for Recognizing Pharmacological Interactions and Virtual Screening
**GPR55**	G protein-coupled receptors 55
**GPR3**	G protein-coupled receptors 3
**NF-κB**	nuclear factor κB
**PPARγ**	peroxisome proliferator-activated receptor γ
**GPCRs**	G protein-coupled receptors
**BBB**	Blood brain barrier
**APP**	Amyloid precursor protein
**GPP**	geranyl diphosphate
**OLA**	olivetolic acid
**NFT**	neurofibilliary tangles
**Aβ**	beta-amyloid
**sAPP-β**	soluble amyloid precursor protein β
**CYP2D6**	Cytochrome P450 2D6
**CYP3A4**	Cytochrome P450 3A4
**P450s**	cytochrome P450 enzymes
**hERG**	human ether-a-go-go-related gene
**LD50**	lethal dose 50
**VDW**	van der Waals interactions
**HB**	hydrogen bonds
**SHY5YAPP^+^ cells**	SH-SY5Y cells transfected with the amyloid precursor protein
**CASTp**	Computed Atlas of Surface Topography of proteins
**Ki**	inhibition constant of enzymes
**Δ** **G**	Gibbs free energy
**TE**	electrostatic forces
**Kcal/mol**	kilo-calorie per mole
**PAINS**	Pan Assay Interference Compounds
**TPSA**	topological molecular polar surface area
**ClogP**	log(c_octanol_/c_water_)
**HIA**	human gastrointestinal absorption
