# Correction: Patil et al. Investigation of *Cannabis sativa* Phytochemicals as Anti-Alzheimer’s Agents: An In Silico Study. *Plants* 2023, *12*, 510

**DOI:** 10.3390/plants15132041

**Published:** 2026-07-01

**Authors:** Nil Patil, Vaishnavi Chandel, Aarzu Rana, Mukul Jain, Prashant Kaushik

**Affiliations:** 1 Department of Life Sciences, Parul Institute of Applied Sciences, Parul University, Vadodara 391760, Gujarat, India; nil.patil22636@paruluniversity.ac.in (N.P.); 211127201088@paruluniversity.ac.in (V.C.); 211127203022@paruluniversity.ac.in (A.R.); 2 Laboratory 209, Cell & Developmental Biology Laboratory, Centre of Research for Development, Parul University, Vadodara 391760, Gujarat, India; 3 Independent Researcher, 46022 Valencia, Spain

In this publication [1], there was an error regarding the affiliation for Prashant Kaushik. The updated affiliation should be as follows: Independent Researcher, 46022 Valencia, Spain.

The authors state that the scientific conclusions are unaffected. This correction was approved by the Academic Editor. The original publication has also been updated.
