# Supplementary material for: Investigation of Cannabis sativa Phytochemicals as Anti-Alzheimer’s Agents: An In Silico Study
Source: Plants (Basel). 2023 Jan 22;12(3):510. doi: 10.3390/plants12030510 (PMC9919841; doi:10.3390/plants12030510)
Supplement: Supplementary file 1 [file plants-12-00510-s001.zip › Supplementary Material.pdf]

Supplementary Materials

Table S1: Overall compounds used in this study

Compounds	Mw	HA	HD	Absorption	Lipinski's rule (Violation)	Solubility	BBB Permeability	CNS Permeability	CYP2D6	HHP
**(-)-CBD**	314.469	2	2	89.308	1	-5.609	-0.074	-1.741	NO	NO
**(+)-CBD**	314.469	4	2	91.162	1	-5.042	0.489	-1.807	NO	NO
**(+)-DMH-CBD**	370.577	2	2	89.034	1	-6.551	-0.208	-1.587	NO	NO
**0-1602**	258.361	2	2	92.957	0	-3.821	0.227	-1.882	NO	NO
**11-HydroxyTHC**	330.468	3	2	91.772	0	-4.367	-0.216	-1.959	NO	NO
**11-Nor-9-carboxy-delta-9 tetrahydrocannabinol**	344.44	3	2	94.696	0	-3.859	-0.367	-2.012	NO	NO
**11-oxo-(delta)9 THC**	328.452	3	1	93.759	0	-4.967	-0.224	-2.017	NO	NO
**2-Aracilonylglycerol**	378.553	4	2	91.67	0	-5.095	-0.238	-2.955	NO	NO
**3-carene**	222.372	1	1	91.645	0	-4.45	0.592	-2.279	NO	NO
**Abn-CBD**	314.469	2	2	90.33	1	-6.075	-0.148	-2.107	NO	NO
**Anandamide**	347.543	2	2	90.907	0	-5.475	-0.516	-2.754	NO	NO
**Apigenin**	270.24	5	3	91.856	0	-3.178	-0.951	-2.211	NO	NO
**Bornyl acetate**	196.29	2	0	95.196	0	-3.009	0.536	-2.376	NO	NO
**Cannabichromanon**	332.44	4	1	93.497	0	-5.055	-0.409	-2.235	NO	NO
**Cannabichromene**	314.469	2	1	90.648	1	-5.532	0.657	-1.976	NO	NO
**Cannabichromenic acid**	358.478	3	2	93.893	0	-3.143	0.107	-2.082	NO	NO
**Cannabichromevarinic acid**	330.424	3	2	94.676	0	-2.972	0.191	-2.003	NO	NO
**Cannabicitran**	314.469	2	0	93.93	1	-5.92	0.379	-2.225	NO	NO
**Cannabicyclol**	314.469	2	1	91.861	1	-5.007	0.551	-1.828	NO	NO
**Cannabicyclovarin**	286.415	2	1	91.271	0	-4.953	0.36	-2.011	NO	NO
**Cannabidibutol**	300.442	2	2	89.528	0	-5.301	-0.025	-1.829	NO	NO
**Cannabidiorcol**	254.329	2	1	92.851	0	-4.599	0.397	-1.368	NO	NO
**Cannabidiphorol**	342.523	2	2	89.555	1	-6.327	-0.196	-1.523	NO	NO
**Cannabidivarinic acid**	330.424	3	3	97.849	0	-3.723	-0.836	-2.016	NO	NO
**Cannabielsoic Acid A**	374.477	4	3	95.774	0	-3.008	-1.01	-2.139	NO	NO
**Cannabielsoic Acid B**	374.477	4	3	97.784	0	-3.507	-0.899	-2.235	NO	NO
**Cannabielsoin**	330.46	5	3	92.665	0	-4.494	-0.2	-1.893	NO	NO
**Cannabifuran**	310.43	2	1	91.801	1	-5.787	-0.14	-1.22	NO	YES
**Cannabigerolic acid**	360.494	3	3	95.782	1	-3.515	-0.911	-2.269	NO	NO
**Cannabigerovarin**	288.431	2	2	90.132	1	-5.533	-0.182	-1.507	NO	NO

**Cannabigerovarinic acid**	332.44	3	3	96.772	0	-3.599	-0.848	-2.276	NO	NO
**Cannabimovone**	332.44	4	3	91.532	0	-3.773	-0.692	-2.317	NO	NO
**Cannabinol**	310.437	2	1	92.888	1	-5.845	0.727	-1.384	NO	NO
**Cannabinol C2**	268.356	2	1	93.96	0	-4.834	0.5	-1.32	NO	YES
**Cannabinol C4**	296.41	2	1	93.14	0	-5.58	0.671	-1.389	NO	NO
**Cannabinol methyl ether**	324.46	2	0	95.747	1	-6.552	0.563	-1.411	NO	NO
**Cannabinolic acid**	354.446	3	2	95.906	0	-3.473	0.176	-1.853	NO	NO
**Cannabiripsol**	348.483	4	3	93.486	0	-3.586	-0.887	-2.105	NO	NO
**Cannabisativine**	381.561	5	4	92.94	0	-2.4	-0.708	-3.47	NO	NO
**Cannabisin A**	594.62	8	8	76.265	2	-2.892	-1.631	-3.339	YES	YES
**Cannabisin B**	596.636	8	8	71.398	2	-2.892	-1.408	-3.472	NO	YES
**Cannabisin C**	610.663	8	7	76.003	2	-2.893	-1.281	-3.377	NO	YES
**Cannabisin D**	624.69	8	6	80.562	2	-2.906	-1.161	-3.263	NO	YES
**Cannabitriol**	346.46	4	3	93.328	0	-3.592	-0.893	-2.094	NO	NO
**Cannabitwinol**	620.789	4	0	100	2	-3.181	-1.125	-0.887	NO	NO
**Cannaflavin A**	436.504	6	3	90.178	0	-4.588	-1.221	-2.084	NO	NO
**Canniprene**	342.435	4	2	92.831	0	-5.563	-0.296	-2.191	NO	NO
**CBD**	314.469	2	2	89.308	1	-5.609	-0.074	-1.741	NO	NO
**CBDA**	358.478	3	3	96.669	0	-3.801	-0.907	-1.993	NO	NO
**CBD-aldehyde-diacetate**	412.526	5	0	95.976	0	-6.556	-0.488	-2.175	NO	NO
**CBD-Q(VIII)**	433.592	4	2	89.931	0	-5.938	-0.203	-1.947	NO	NO
**CBDV**	286.415	2	2	89.745	0	-5.099	-0.003	-1.901	NO	NO
**CBE**	235.308	3	1	92.597	0	-2.897	0.236	-1.97	NO	YES
**CBG**	316.485	2	2	87.518	1	-4.687	-0.302	-0.86	NO	NO
**Chrysoeriol**	300.266	6	3	85.342	0	-3.38	-1.046	-2.419	NO	NO
**Dehydrocannabifuran**	308.421	2	1	90.563	1	-5.693	0.035	-1.158	NO	YES
**Delta 8 THC**	314.469	2	1	92.205	1	-5.374	0.493	-1.865	NO	NO
**Delta-9-Cannabivarinic Acid**	330.424	3	2	94.903	0	-2.956	0.295	-1.778	NO	NO
**Delta-9-cis-tetrahydrocannabinol**	314.469	2	1	91.162	1	-5.042	0.489	-1.807	NO	NO
**Delta-9-tetrahydrocannabinolic acid**	358.478	3	2	94.353	0	-3.137	0.199	-1.748	NO	NO
**Delta-9-tetrahydrocannabiorcol**	258.361	2	1	91.943	0	-3.683	0.21	-2.163	NO	NO
**Delta-guaiene**	204.357	0	0	93.329	1	-5.99	0.773	-1.838	NO	NO
**Eucalyptol**	154.253	1	0	96.505	0	-2.63	0.368	-2.972	NO	NO

**H2 DMH-CBD**	372.593	2	2	89.242	1	-6.29	-0.401	-1.202	NO	NO
**HU-308**	414.63	3	1	93.478	1	-6.512	-0.209	-2.255	NO	NO
**HU-331**	328.452	3	1	94.314	0	-4.62	-0.004	-1.94	NO	NO
**HUF-101**	332.459	2	2	88.362	1	-5.21	0.029	-1.912	NO	NO
**KLS-13019**	355.47	3	2	91.629	0	-4.284	-0.186	-1.976	NO	YES
**THC**	314.469	2	1	91.162	1	-5.042	0.489	-1.807	NO	NO
**THCA-B**	358.478	3	2	94.833	0	-3.645	-0.357	-1.839	NO	NO
**THC-acetate**	356.506	3	0	93.655	1	-6.244	0.197	-2.23	NO	NO
**THC-COOH**	344.451	3	2	94.696	0	-3.859	-0.367	-2.012	NO	NO
**THCV**	286.415	2	1	91.821	0	-4.403	0.336	-1.99	NO	NO
**VCE-003.2**	373.537	4	2	90.024	0	-5.117	-0.337	-2.33	NO	NO
**VCE-004.8**	433.592	4	2	89.931	0	-5.938	-0.203	-1.947	NO	NO

Table S2: Compounds passing HHP and Lipinski's rule

**Compound**	**m.w**	**HA**	**HD**	**Absorption**	**Lipinski's rule (Violation)**	**Solubility**	**BBB Permeability**	**CNS Permeability**	**CYP2D6**
0-1602	258.361	2	2	92.957	0	-3.821	0.227	-1.882	NO
11-HydroxyTHC	330.468	3	2	91.772	0	-4.367	-0.216	-1.959	NO
11-Nor-9-carboxy-delta-9 tetrahydrocannabinol	344.44	3	2	94.696	0	-3.859	-0.367	-2.012	NO
11-oxo-(delta)9 THC	328.452	3	1	93.759	0	-4.967	-0.224	-2.017	NO
3-carene	222.372	1	1	91.645	0	-4.45	0.592	-2.279	NO
Apigenin	270.24	5	3	91.856	0	-3.178	-0.951	-2.211	NO
Bornyl acetate	196.29	2	0	95.196	0	-3.009	0.536	-2.376	NO
Cannabichromanon	332.44	4	1	93.497	0	-5.055	-0.409	-2.235	NO
Cannabichromenic acid	358.478	3	2	93.893	0	-3.143	0.107	-2.082	NO
Cannabichromevarinic acid	330.424	3	2	94.676	0	-2.972	0.191	-2.003	NO
Cannabicyclovarin	286.415	2	1	91.271	0	-4.953	0.36	-2.011	NO
Cannabidibutol	300.442	2	2	89.528	0	-5.301	-0.025	-1.829	NO
Cannabidiorcol	254.329	2	1	92.851	0	-4.599	0.397	-1.368	NO
Cannabidivarinic acid	330.424	3	3	97.849	0	-3.723	-0.836	-2.016	NO
Cannabielsoic Acid A	374.477	4	3	95.774	0	-3.008	-1.01	-2.139	NO
Cannabielsoic Acid B	374.477	4	3	97.784	0	-3.507	-0.899	-2.235	NO
Cannabielsoin	330.46	5	3	92.665	0	-4.494	-0.2	-1.893	NO
Cannabigerovarinic acid	332.44	3	3	96.772	0	-3.599	-0.848	-2.276	NO

Cannabimovone	332.44	4	3	91.532	0	-3.773	-0.692	-2.317	NO
Cannabinol C2	268.356	2	1	93.96	0	-4.834	0.5	-1.32	NO
Cannabinol C4	296.41	2	1	93.14	0	-5.58	0.671	-1.389	NO
Cannabinolic acid	354.446	3	2	95.906	0	-3.473	0.176	-1.853	NO
Cannabiripsol	348.483	4	3	93.486	0	-3.586	-0.887	-2.105	NO
Cannabisativine	381.561	5	4	92.94	0	-2.4	-0.708	-3.47	NO
Cannabitriol	346.46	4	3	93.328	0	-3.592	-0.893	-2.094	NO
Cannaflavin A	436.504	6	3	90.178	0	-4.588	-1.221	-2.084	NO
Canniprene	342.435	4	2	92.831	0	-5.563	-0.296	-2.191	NO
CBDA	358.478	3	3	96.669	0	-3.801	-0.907	-1.993	NO
CBD-aldehyde-diacetate	412.526	5	0	95.976	0	-6.556	-0.488	-2.175	NO
CBD-Q(VIII)	433.592	4	2	89.931	0	-5.938	-0.203	-1.947	NO
CBDV	286.415	2	2	89.745	0	-5.099	-0.003	-1.901	NO
CBE	235.308	3	1	92.597	0	-2.897	0.236	-1.97	NO
Chrysoeriol	300.266	6	3	85.342	0	-3.38	-1.046	-2.419	NO
Delta-9-Cannabivarinic Acid	330.424	3	2	94.903	0	-2.956	0.295	-1.778	NO
Delta-9-tetrahydrocannabinolic acid	358.478	3	2	94.353	0	-3.137	0.199	-1.748	NO
Delta-9-tetrahydrocannabiorcol	258.361	2	1	91.943	0	-3.683	0.21	-2.163	NO
Eucalyptol	154.253	1	0	96.505	0	-2.63	0.368	-2.972	NO
HU-331	328.452	3	1	94.314	0	-4.62	-0.004	-1.94	NO
KLS-13019	355.47	3	2	91.629	0	-4.284	-0.186	-1.976	NO
THCA-B	358.478	3	2	94.833	0	-3.645	-0.357	-1.839	NO
THC-COOH	344.451	3	2	94.696	0	-3.859	-0.367	-2.012	NO
THCV	286.415	2	1	91.821	0	-4.403	0.336	-1.99	NO
VCE-003.2	373.537	4	2	90.024	0	-5.117	-0.337	-2.33	NO
VCE-004.8	433.592	4	2	89.931	0	-5.938	-0.203	-1.947	NO

Table S3: Compounds passing BBB permeability

Compounds	Absorption	Lipinski's rule (Violation)	Solubility	BBB Permeability	CNS Permeability	CYP2D6	LD50	Toxicity class
**3-carene**	91.645	0	-4.45	0.592	-2.279	NO	2000	4

**Bornyl acetate**	95.196	0	-3.009	0.536	-2.376	NO	3100	5
**Cannabicyclovarin**	91.271	0	-4.953	0.36	-2.011	NO	860	4
**Cannabidiorcol**	92.851	0	-4.599	0.397	-1.368	NO	800	4
**Cannabinol C2**	93.96	0	-4.834	0.5	-1.32	NO	1310	4
**Eucalyptol**	96.505	0	-2.63	0.368	-2.972	NO	2480	5
**THCV**	91.821	0	-4.403	0.336	-1.99	NO	482	4
