# Supplementary figures and images for: Investigation of Cannabis sativa Phytochemicals as Anti-Alzheimer’s Agents: An In Silico Study
Source: Plants (Basel). 2023 Jan 22;12(3):510. doi: 10.3390/plants12030510 (PMC9919841; doi:10.3390/plants12030510)

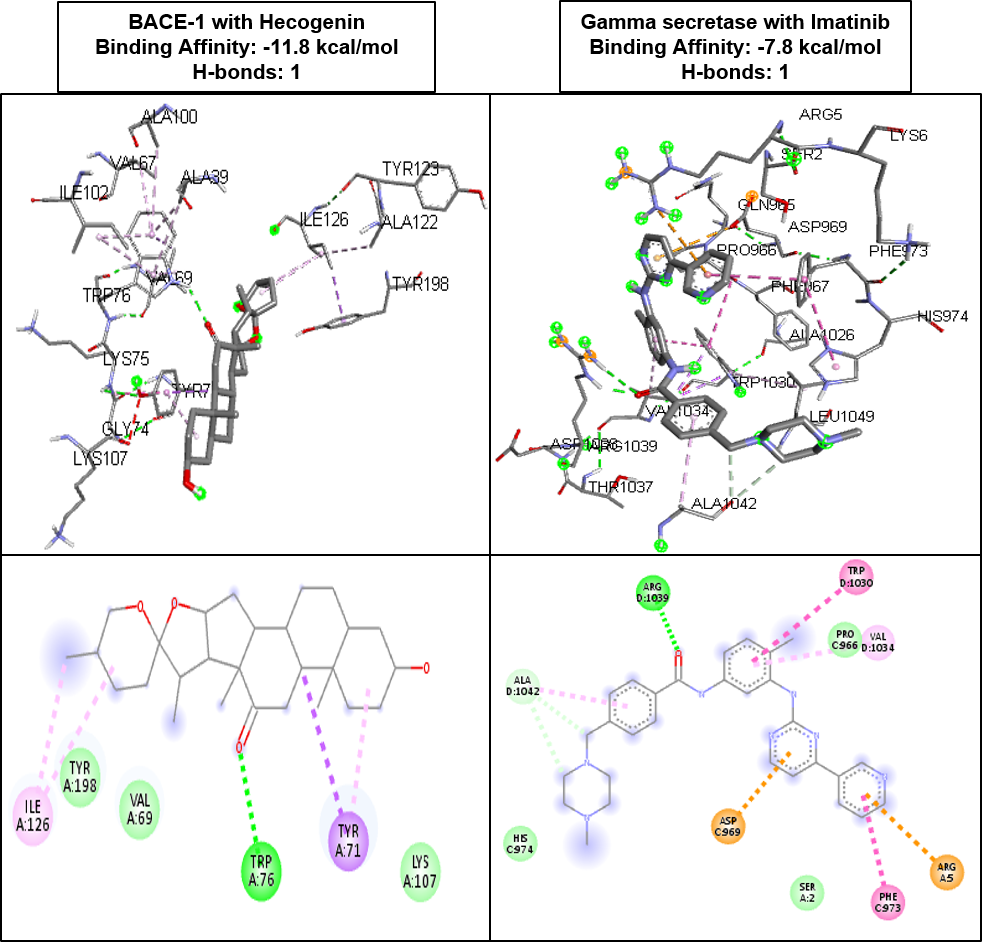

Supplement: Supplementary file 1 [file plants-12-00510-s001.zip › Supplementary Figure S1.tif]
